# Supplementary material for: The ubiquitin-like modifier FAT10 covalently modifies HUWE1 and strengthens the interaction of AMBRA1 and HUWE1
Source: PLoS One. 2023 Aug 14;18(8):e0290002. doi: 10.1371/journal.pone.0290002 (PMC10424871; doi:10.1371/journal.pone.0290002)
Supplement: S1 File — (PDF) [file pone.0290002.s001.pdf]

Supporting information

The ubiquitin-like modifier FAT10 covalently modifies HUWE1 and strengthens the interaction of AMBRA1 and HUWE1

Stefanie Mueller[1,2], Johanna Bialas[1,2], Stella Ryu[1,2], Nicola Catone[1] and Annette Aichem[1,2*]

[1]Biotechnology Institute Thurgau at the University of Konstanz, Kreuzlingen, Switzerland

[2]Division of Immunology, Department of Biology, University of Konstanz, Germany

[*]Correspondence: Annette.Aichem@bitg.ch; Tel.: +41716785034

Short title: FAT10ylation of HUWE1 targets it for proteasomal degradation

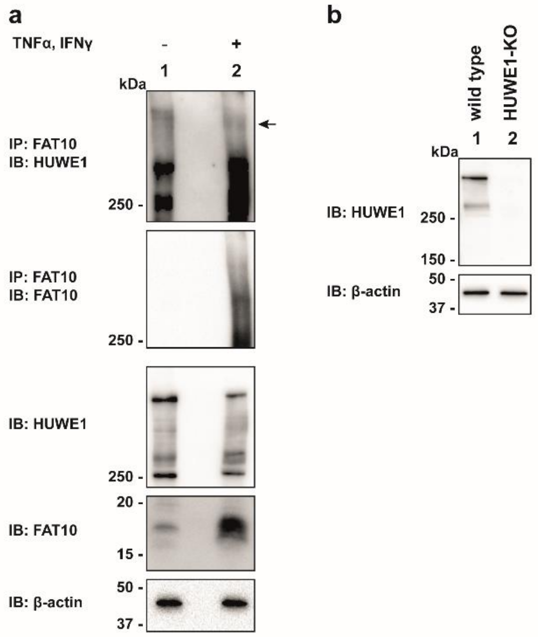

S1 Fig. Detection of endogenous HUWE1-FAT10 conjugate. (a) HEK293 wild type cells were treated with TNF and IFN-γ for 24 hours to induce endogenous expression of FAT10, which was then immunoprecipitated with a monoclonal anti-FAT10 (4F1) antibody coupled to protein A sepharose. Samples were subjected to SDS-PAGE followed by Western blot analysis. Proteins were visualized with antibodies against HUWE1 (Mule), FAT10, and β-actin as loading control. One experiment out of three experiments with similar outcomes is shown. (b) HEK293 HUWE1-KO cells were generated via CRISPR-Cas9-mediated gene inactivation. Successful knockout of single cell clones was confirmed with anti-HUWE1 (Mule) antibody.

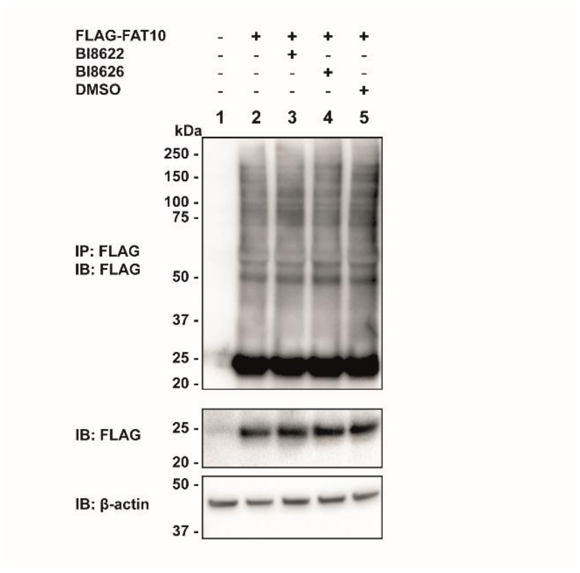

S2 Fig. Pharmacological inhibition of HUWE1 does not impair bulk FAT10 conjugation. HEK293 wild type cells were transfected with an expression plasmid for FLAG-FAT10. After 24 hours, cells were treated with either one of the HUWE1-inhibitors BI8622 and BI8626 (10 µM) [59]or DMSO as a control for six hours before cell lysis, immunoprecipitation and Western blot analysis. Subsequently, FAT10 conjugates were analyzed with antibodies against the FLAG-tag or β-actin as loading control. The experiment was performed twice with similar outcomes.

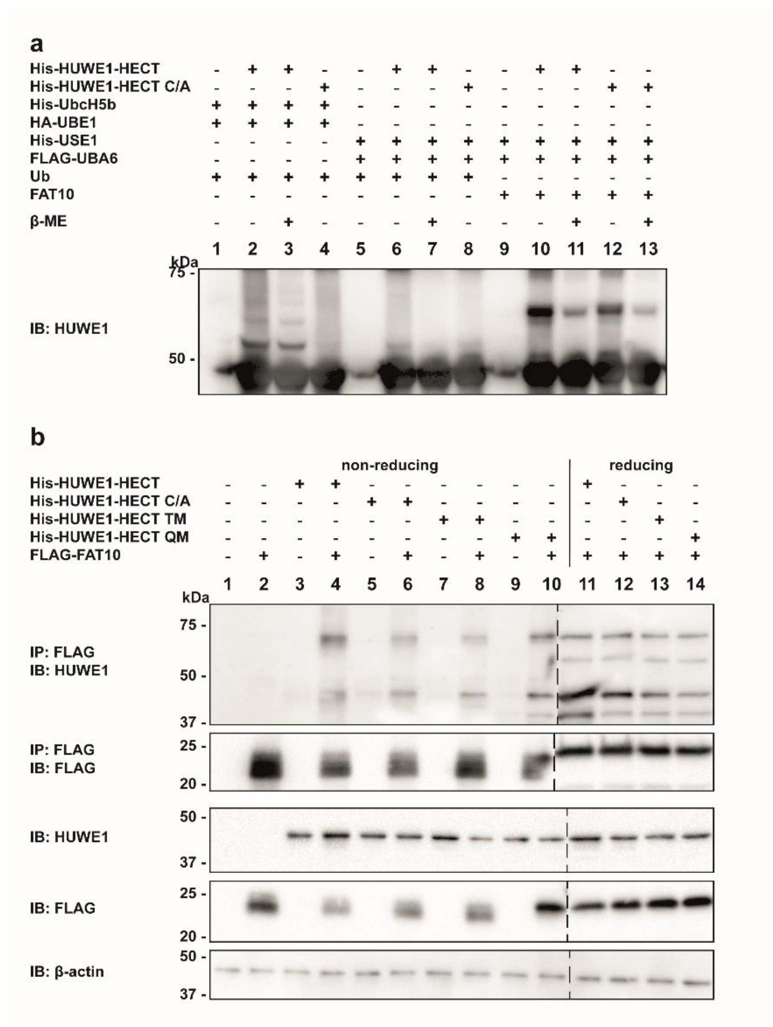

S3 Fig. ***In vitro*** **transfer of ubiquitin or FAT10 onto HUWE1 variants.** (a) To determine the interaction between ubiquitin and HUWE1-HECT wild type or its active site cysteine mutant, i*n vitro* assays were performed with recombinant cysteine variants of 6His-HUWE1-HECT (amino acid residues 3993 – 4374). Proteins were incubated with recombinant 6His-UbcH5b, HA-UBE1 or 6His-USE1, FLAG-UBA6 and tagless ubiquitin or FAT10, as indicated, at 37 °C for 30 min. Samples were analyzed under non-reducing and reducing (4 % β-ME) conditions by SDS-PAGE and Western blot analysis using an anti-HUWE1 (ARF-BP1) antibody. One representative experiment out of two with similar outcomes is shown. (b) HEK293 HUWE1-KO cells were transfected with plasmids expressing FLAG-FAT10 and different His-tagged HUWE1-HECT cysteine variants. After 24 hours, co-immunoprecipitation from cleared lysates was performed using Anti-FLAG M2 Affinity Gel. Samples were subjected under non-reducing or reducing conditions (4% β-ME) to SDS-PAGE and subsequent Western blotting using antibodies reactive against HUWE1 (ARF-BP1) and FLAG, as well as anti-β-actin as loading control. Shown is one representative experiment out of three with similar outcomes.

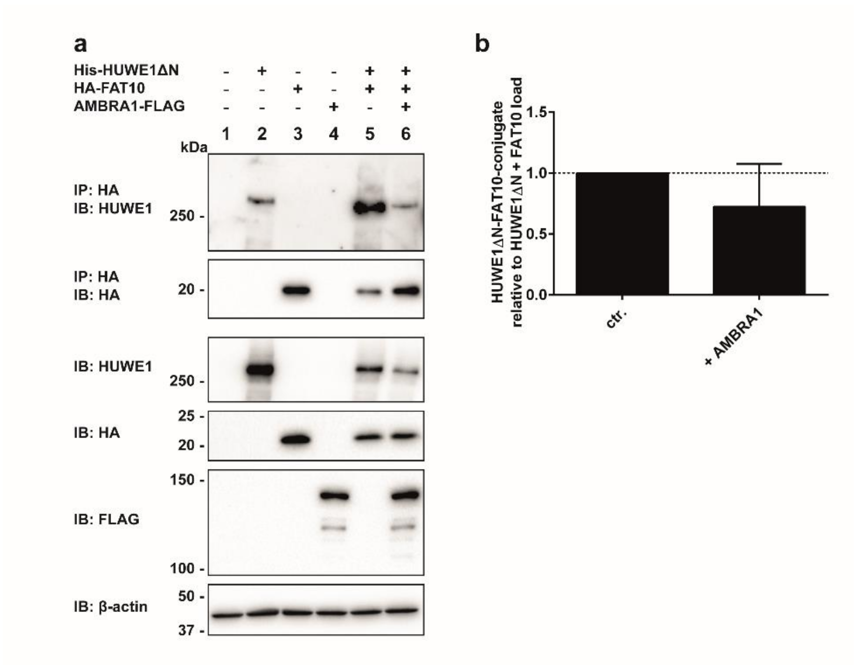

S4 Fig. The amount of the HUWE1-FAT10 conjugate is diminished in presence of AMBRA1. (a) Western blot from co-immunoprecipitation experiments of His-HUWE1ΔN and HA-FAT10 in the presence of AMBRA1-FLAG using cleared lysates of HEK293 HUWE1-KO cells which were transfected with the corresponding expression plasmids. Displayed is one representative experiment out of three with comparable outcomes together with the quantification (b) from densitometric analysis of Western blot signals shown in (a) as described above.
