# Supplementary material for: The ubiquitin-like modifier FAT10 covalently modifies HUWE1 and strengthens the interaction of AMBRA1 and HUWE1
Source: PLoS One. 2023 Aug 14;18(8):e0290002. doi: 10.1371/journal.pone.0290002 (PMC10424871; doi:10.1371/journal.pone.0290002)

raw images_Figure 1

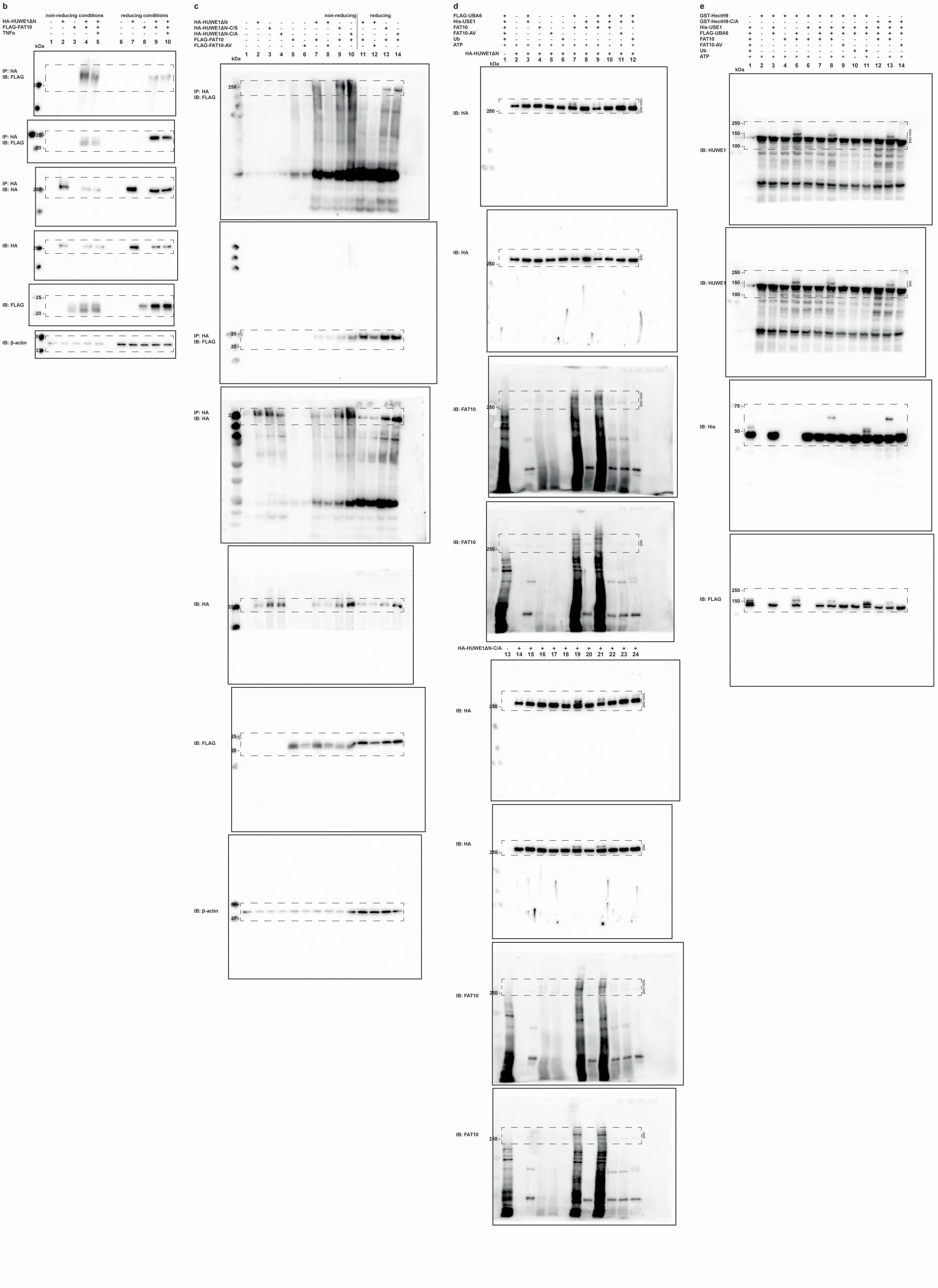

raw images_Figure 2

a

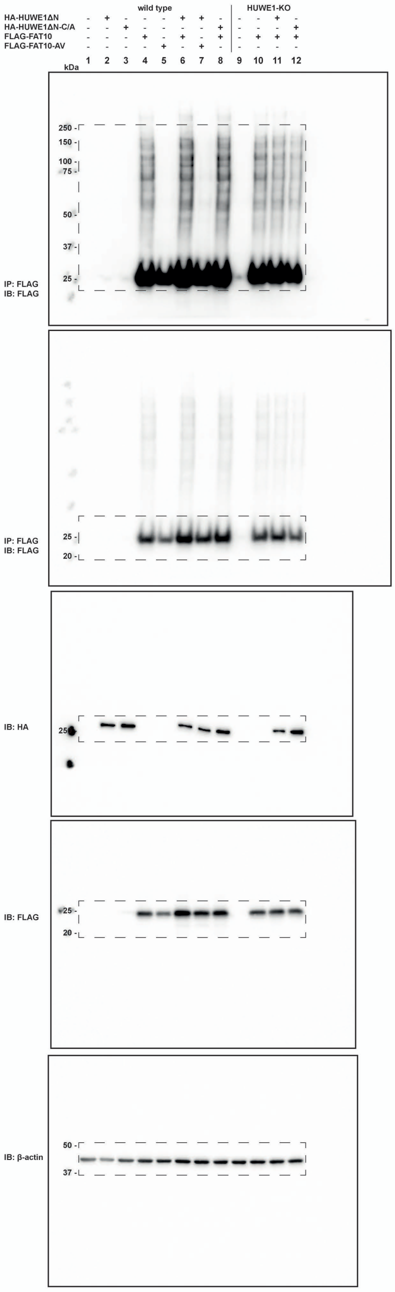

c

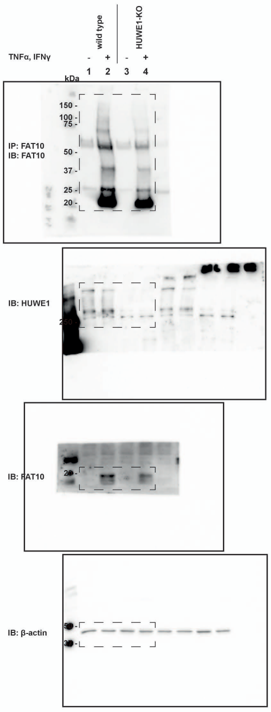

raw images_Figure 3

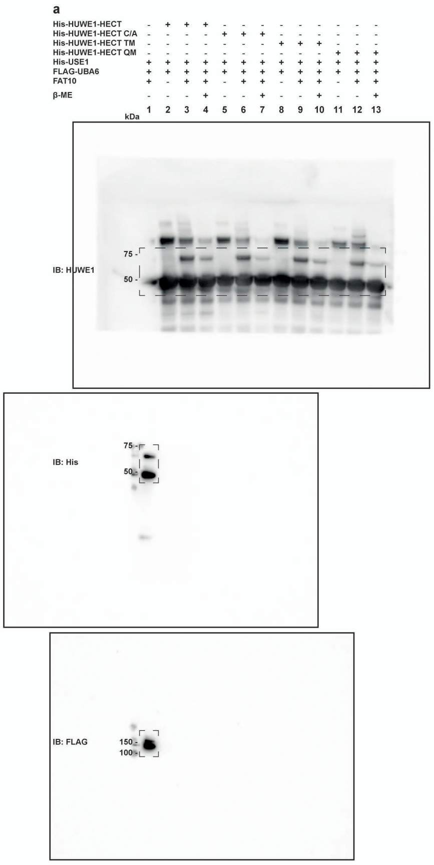

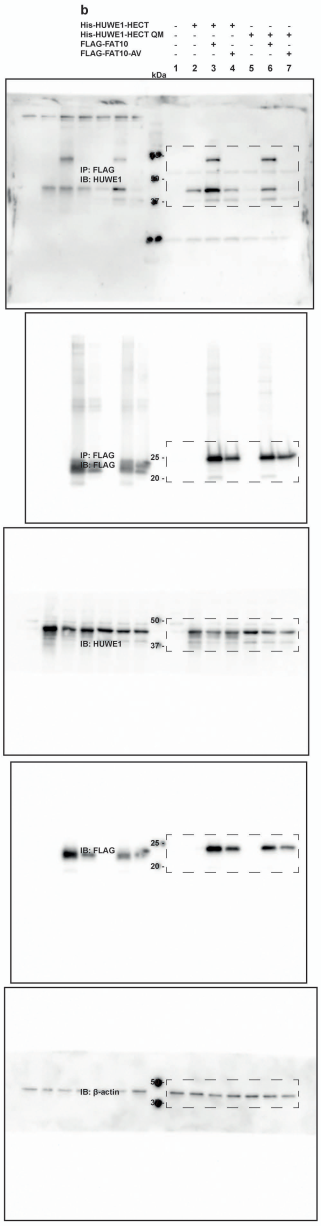

raw images_Figure 4

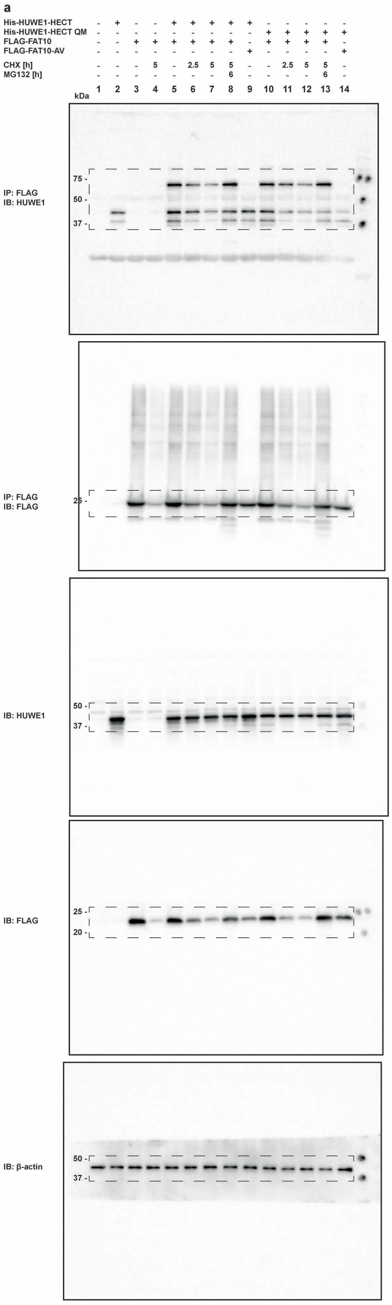

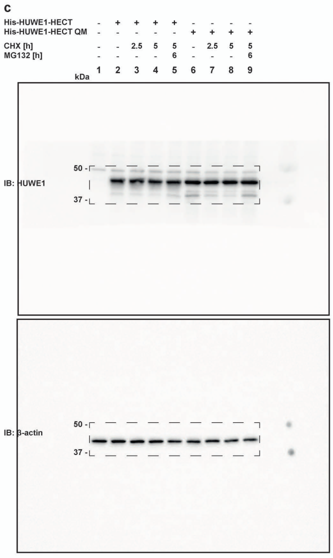

raw images_Figure 5

a

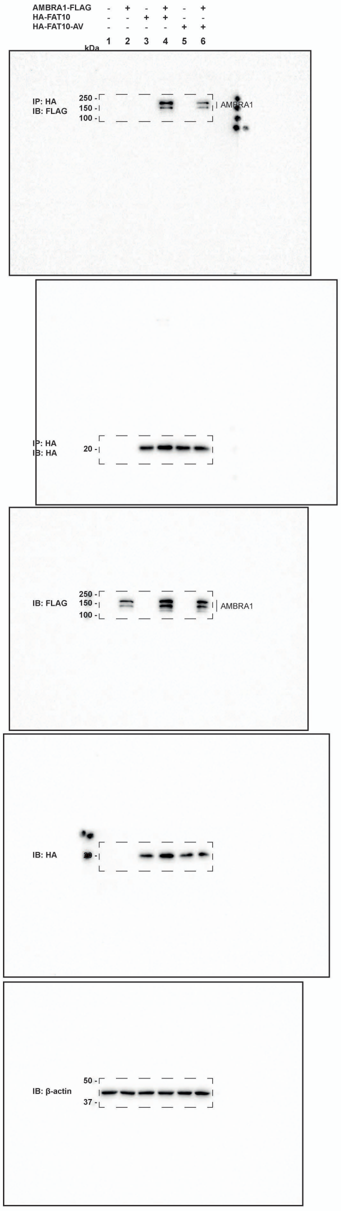

b

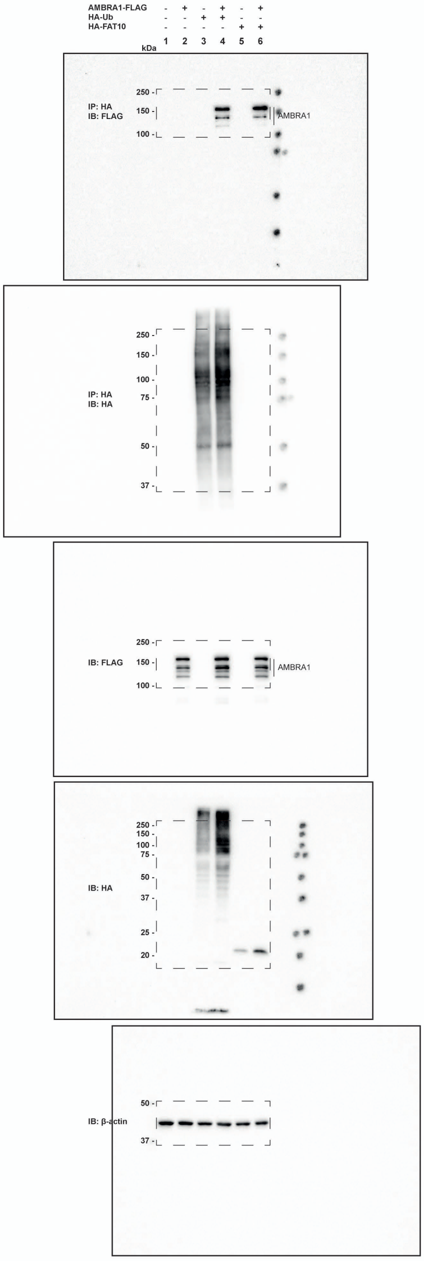

c

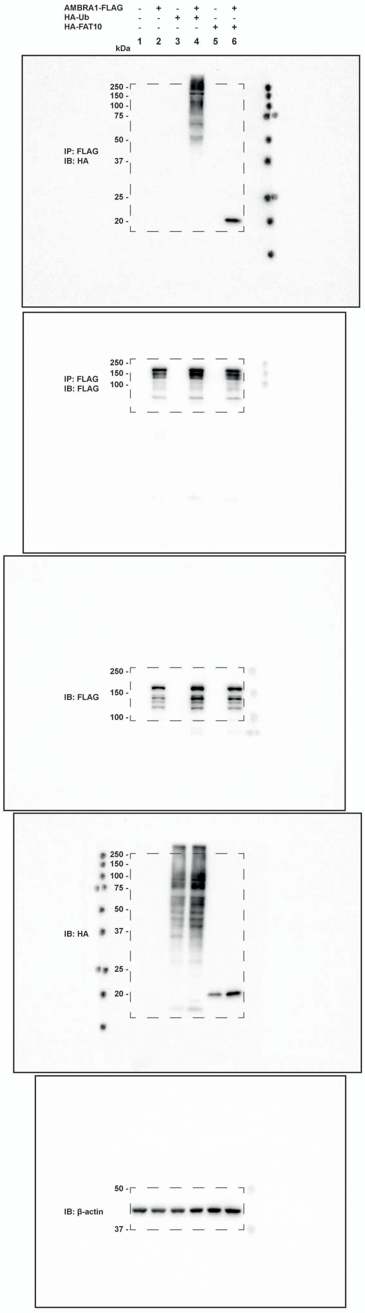

raw images_Figure 6

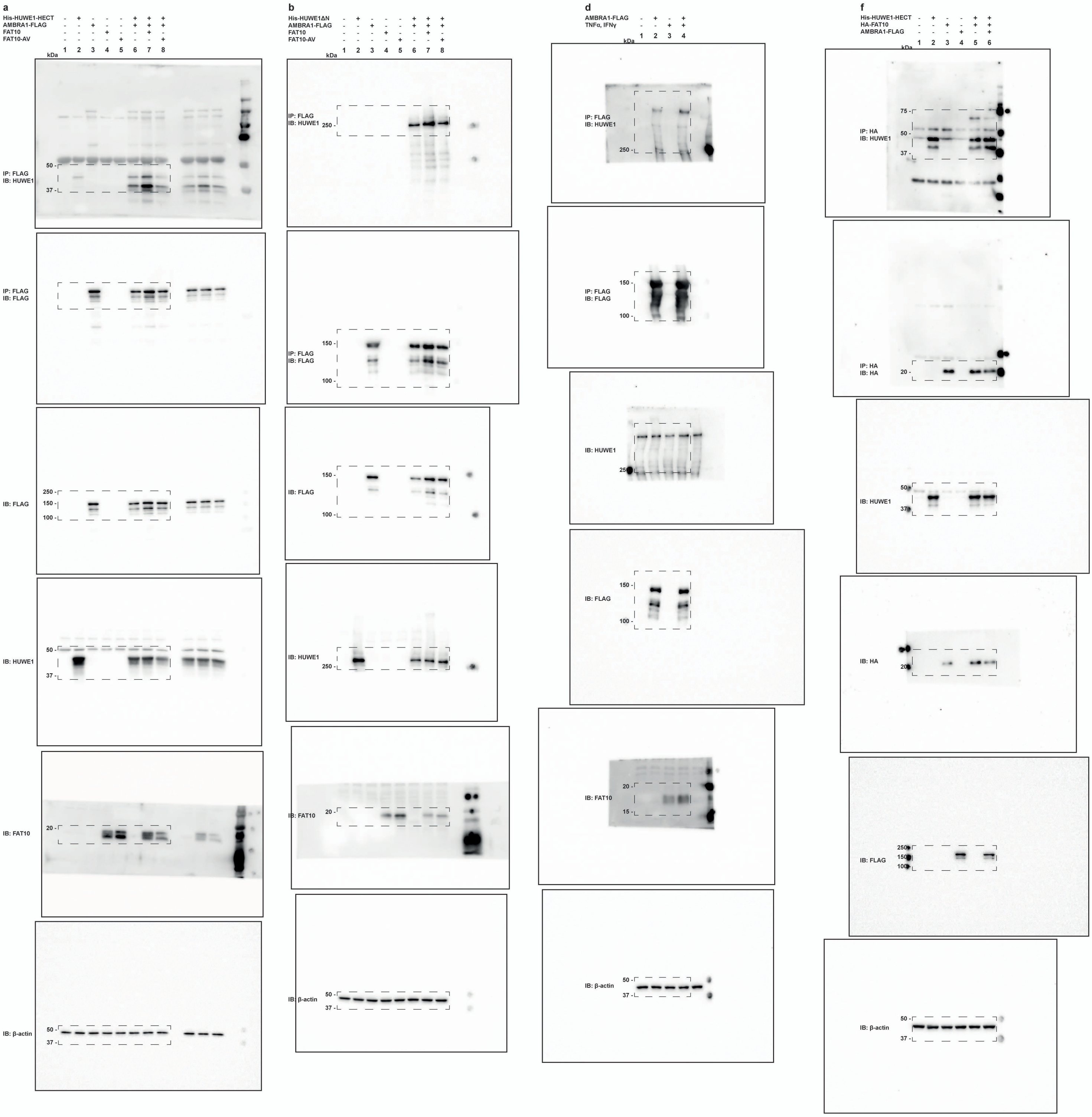

raw images_Figure S1

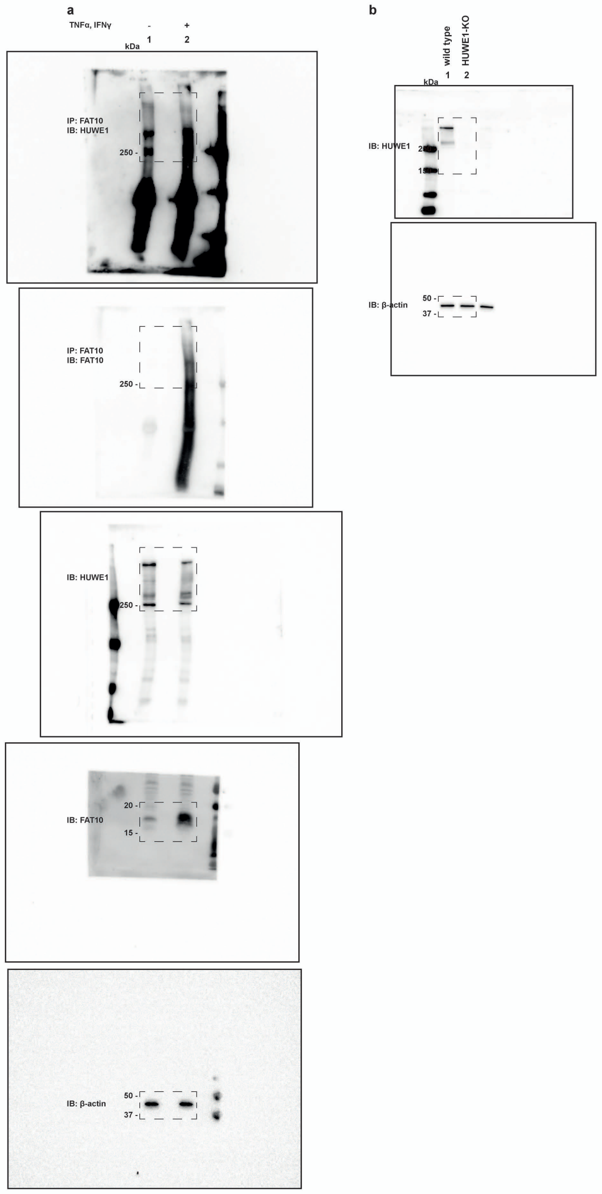

raw images_Figure S2

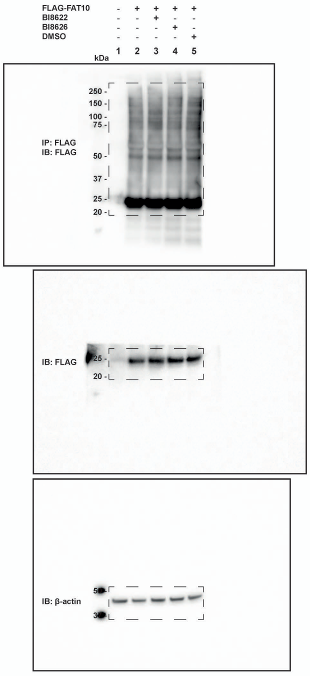

raw images_Figure S3

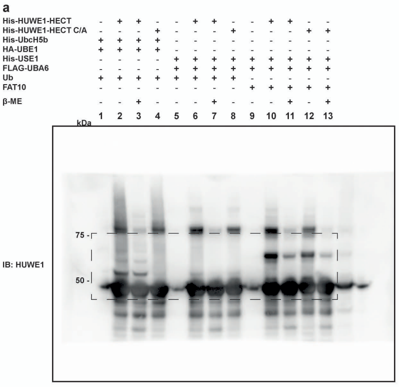

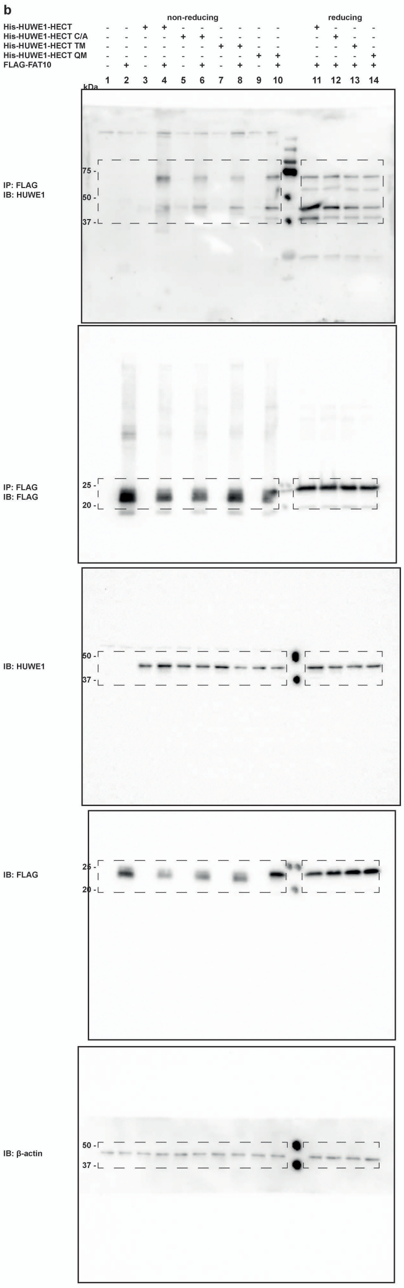

raw images_Figure S4

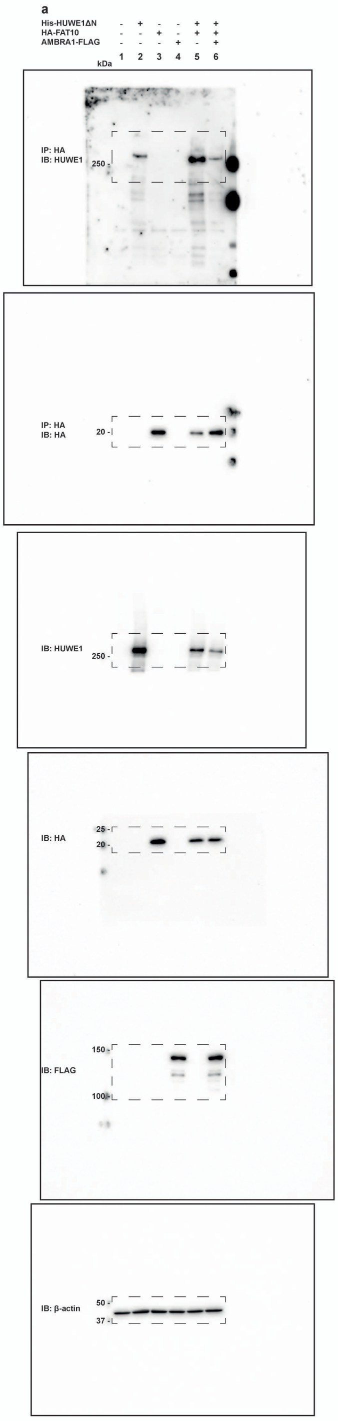

Supplement: S1 Raw images — (PDF) [file pone.0290002.s002.pdf]
